# Perspective on Aqueous Batteries: Historical Milestones and Modern Revival

**DOI:** 10.1002/adma.72294

**Published:** 2026-02-03

**Authors:** Fangwang Ming, Dong Guo, Yizhou Wang, Hussam Qasem, Hanfeng Liang, Husam N. Alshareef

**Affiliations:** ^1^ Center for Renewable Energy and Storage Technologies (CREST) King Abdullah University of Science and Technology (KAUST) Thuwal Saudi Arabia; ^2^ Materials Science and Engineering Physical Science and Engineering (PSE) Division King Abdullah University of Science and Technology (KAUST) Thuwal Saudi Arabia; ^3^ Future Energy Technology Institute King Abdulaziz City for Science and Technology (KACST) Riyadh Saudi Arabia; ^4^ State Key Laboratory of Physical Chemistry of Solid Surfaces College of Chemistry and Chemical Engineering Xiamen University Xiamen China

**Keywords:** aqueous batteries, historical evolution, Ni‐based batteries, Zn based batteries

## Abstract

Aqueous batteries have played a pivotal yet fluctuating role in the evolution of electrochemical energy storage. From their foundational success in lead–acid and nickel‐based chemistries to their eclipse by lithium‐ion batteries, aqueous systems were long regarded as technologically inferior due to limited energy density and poor cycling stability. However, the urgent demand for safe, low‐cost, and sustainable storage has sparked a renaissance, fueled by breakthroughs in electrolyte engineering and advanced electrode materials for both anodes and cathodes. This review revisits the historical trajectory of commercialized aqueous batteries, extracting lessons from past successes and failures while highlighting the technological advances that now enable extended voltage windows, improved cycling stability, and scalable manufacturing. We argue that the future of aqueous batteries lies not in directly competing with lithium‐ion in high‐energy applications, but in complementing them across grid‐scale storage, uninterruptible power supplies, and decentralized energy systems where safety, cost, and recyclability are paramount. By connecting history with current progress, we reflect on how these insights reshape expectations for the next generation of aqueous batteries and their role in a more diversified and sustainable energy storage landscape.

## Graphical abstract

This review retraces the development of aqueous batteries from classical Zn‐MnO_2_ chemistry to modern Zn and Ni systems, correlating voltage, capacity, and electrolyte formulation with practical performance. By mapping historical success and failure onto current and future research directions, it identifies guiding principles that steer the design of safe, low‐cost, and application‐oriented aqueous energy storage.

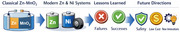

## 1 Introduction

The story of battery technology is one of continuous evolution, driven by the quest for reliable, efficient, and portable energy storage. From the rudimentary voltaic pile of Alessandro Volta in 1800 [1] to the sophisticated electrochemical systems of the 21st century, batteries have transformed how we power our world [2, 3, 4]. The earliest practical batteries, such as the lead‐acid system invented by Gaston Planté in 1859 [5, 6], marked the beginning of rechargeable energy storage, enabling applications from industrial machinery to early automobiles. Over the subsequent century, nickel‐based chemistries (e.g., nickel‐cadmium (Ni‐Cd) and nickel‐metal hydride (Ni‐MH)) emerged [7, 8, 9], offering improved performance and powering portable devices and electric vehicles (EVs). However, the landscape of battery technology shifted dramatically with the advent of lithium‐ion batteries (LIBs) in the late 20th century [2, 3, 4]. First commercialized by Sony in 1991, LIBs capitalized on the lightweight nature of lithium, the smallest metallic element, and the use of organic electrolytes to achieve unprecedented energy densities of 250–300 Wh/kg and nominal voltages exceeding 3.7 V. This breakthrough made LIBs the cornerstone of modern portable electronics, EVs, and renewable energy storage systems [10, 11, 12]. Their dominance stems not only from technical superiority (high energy density, long cycle life, and versatility) but also from massive investments and economies of scale driven by consumer demand and industrial innovation. Today, LIBs are ubiquitous, powering everything from true wireless earbuds, smartphones, drones to EVs, and are integral to the global transition to clean energy.

Amid this evolution, aqueous batteries represent a compelling yet checkered chapter. In their early days, aqueous systems like lead‐acid [5, 6], Ni‐Cd [7], and Ni‐MH [8] offered a compelling promise: safe and cost‐effective energy storage. The non‐flammable nature of water eliminated the fire hazards associated with organic solvents, while abundant materials like lead, zinc, and nickel kept costs low (Figure 6). Lead‐acid batteries, for instance, became the backbone of automotive starting systems and stationary power backups due to their robustness and affordability. Ni‐Cd and Ni‐MH batteries found niches in military applications and consumer electronics, leveraging their durability and rechargeability. Yet, these systems faced significant setbacks that curtailed their broader adoption. The electrochemical stability window of water, limited to approximately 1.23 V due to the decomposition of water into hydrogen and oxygen, capped their energy density at around 100 Wh/kg (especially for rechargeable batteries), far below LIBs. Additionally, issues like structural degradation (e.g., sulfation in lead‐acid batteries [13]) and poor cycling stability restricted their lifespan and performance [14]. As the demand for compact, high‐energy solutions grew in the 1990s, aqueous batteries struggled to compete, losing ground to the superior metrics of LIBs and becoming relegated to niche roles like grid backup and low‐power applications.

**FIGURE 1:**
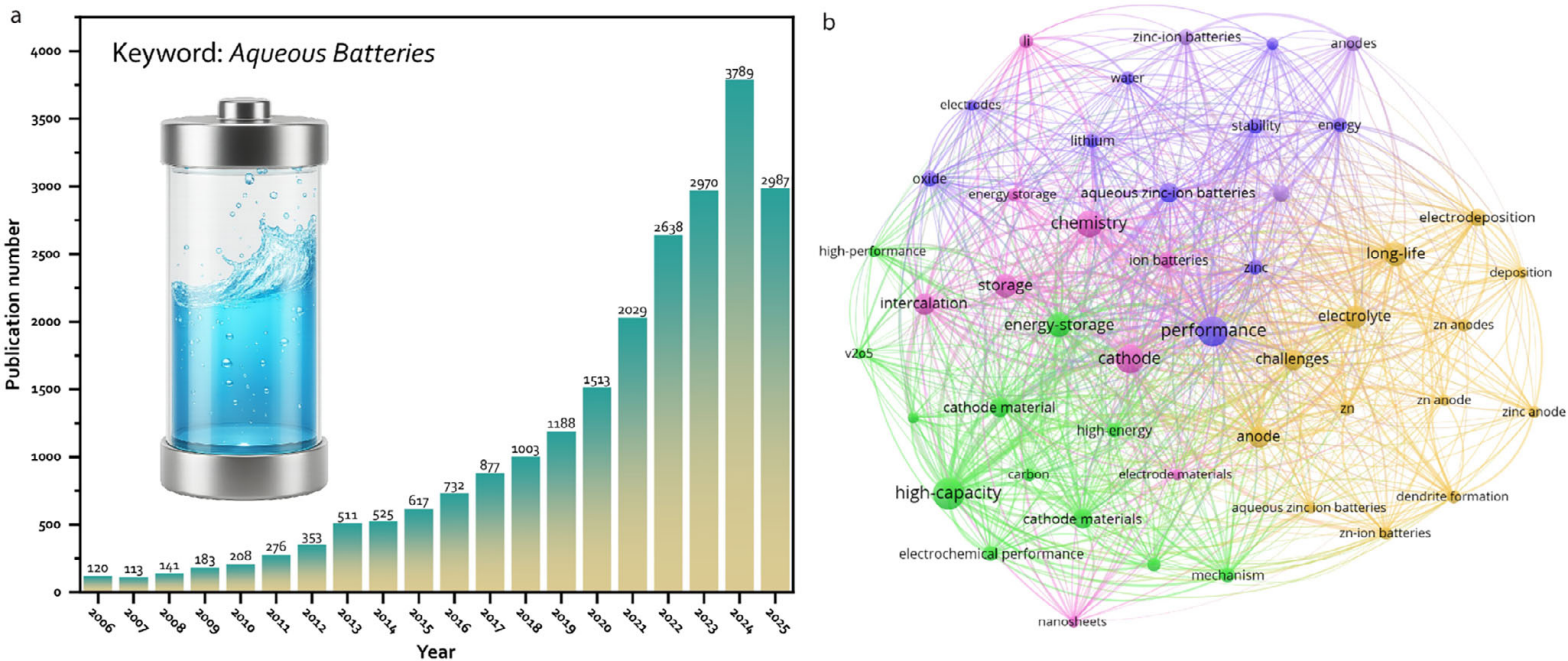
(a) Publication number of “Aqueous batteries” over the past 20 years. (b) Keywords Co‐occurrence network visualization map (Source: Web of Science, Clarivate (retrieved and analyzed on November 7, 2025).

Despite their historical decline, aqueous batteries are experiencing a resurgence of interest in the contemporary energy landscape (see recent publication trend in Figure 1), driven by pressing global challenges and technological advancements. The dominance of LIBs, while transformative, has exposed vulnerabilities: their flammable electrolytes pose safety risks, as evidenced by high‐profile EV fires [15, 16, 17], while their reliance on scarce resources like lithium and cobalt raises concerns about supply chain sustainability and cost [18, 19, 20]. Moreover, the environmental toll of LIB production and disposal, requiring energy‐intensive processes and complex recycling, clashes with the goals of a circular economy. Aqueous batteries, with their inherent safety, use of abundant materials, and simpler recycling potential, offer a counterpoint to these issues [21, 22]. The rise of renewable energy sources, such as solar and wind [23], has further amplified their relevance, as grid‐scale energy storage demands solutions that prioritize safety, scalability, and longevity over the high energy density needed for portable devices. Recent innovations, such as water‐in‐salt electrolytes (WiSEs) [24] that expand the voltage window to ∼3 V and advanced electrode materials that enhance stability [14, 25], are addressing the historical limitations of aqueous systems. These developments, coupled with a global push for sustainable technologies, have sparked renewed research and industrial efforts to reimagine aqueous batteries as viable competitors. Today, as the world seeks alternatives to complement LIBs, aqueous batteries stand at a crossroads, poised to leverage their historical strengths and modern breakthroughs to reclaim a significant role in the future of energy storage.

Although numerous review articles on aqueous batteries have appeared in recent years, many primarily summarize existing research without offering new conceptual insights. Such compilations provide valuable literature surveys but often fall short of advancing fundamental understanding or guiding future research. This review, however, distinguishes itself by two key observations. First, while aqueous battery research has rapidly grown, many researchers are not fully aware of the deep historical roots. Much of the current progress reflects ideas first explored decades or even centuries ago, yet the field rarely revisits these origins. We believe that understanding this history offers essential lessons that can help avoid merely recycling old concepts without real progress. Second, unlike conventional reviews, our work is structured around a historical framework, critically analyzing past successes and failures to offer direction for future research. We revisit the evolution of commercial aqueous batteries not to merely recount their historical trajectory but to extract enduring principles that shaped their emergence, success, and decline. By tracing the advancement and setbacks of Zn‐based and Ni‐based chemistries in response to changing performance demands, we highlight the fundamental constraints and opportunities that continue to influence aqueous systems today.

This historical lens also sets up a natural bridge to the present: Many of the challenges facing modern batteries, including interfacial instability, electrolyte compatibility, and structural degradation, echo problems first recognized more than a century ago. At the same time, contemporary research on lithium metal and LIBs provides valuable reverse guidance, offering conceptual and mechanistic insights that can inform the stabilization of metal anodes, the design of robust interphases, and the optimization of ion transport in aqueous environments. Building on this dual perspective, we connect historical lessons with recent progress in electrolyte engineering, electrode innovation, and device‐level design to outline research directions with the greatest potential for durable and application‐aligned impact. Through this framework, we aim to position current aqueous battery development within a broader technological continuum and clarify how both past experience and modern advances can jointly guide the next stage of innovation.

The development of aqueous batteries can be broadly divided into four evolutionary stages. (1) The foundational period, beginning with the Voltaic pile and culminating in the lead‐acid battery, established the electrochemical basis for subsequent battery systems. (2) The rise and refinement of Zn–MnO_2_ chemistries, initiated by the Leclanché cell, led to widespread adoption of dry cells, ZnCl_2_ cells, and ultimately the high‐performance alkaline battery, which remains commercially dominant in primary applications. (3) The diversification into Zn–X systems, which retained Zn as the anode while exploring alternative cathodes, aimed to increase energy density and, in some cases, rechargeability. (4) The development of Ni–Y rechargeable chemistries, centered on NiOOH cathodes paired with different anodes, prioritized improvements in energy density and cycle life.

## 2 The Early Days: Inception and Initial Success

**FIGURE 2:**
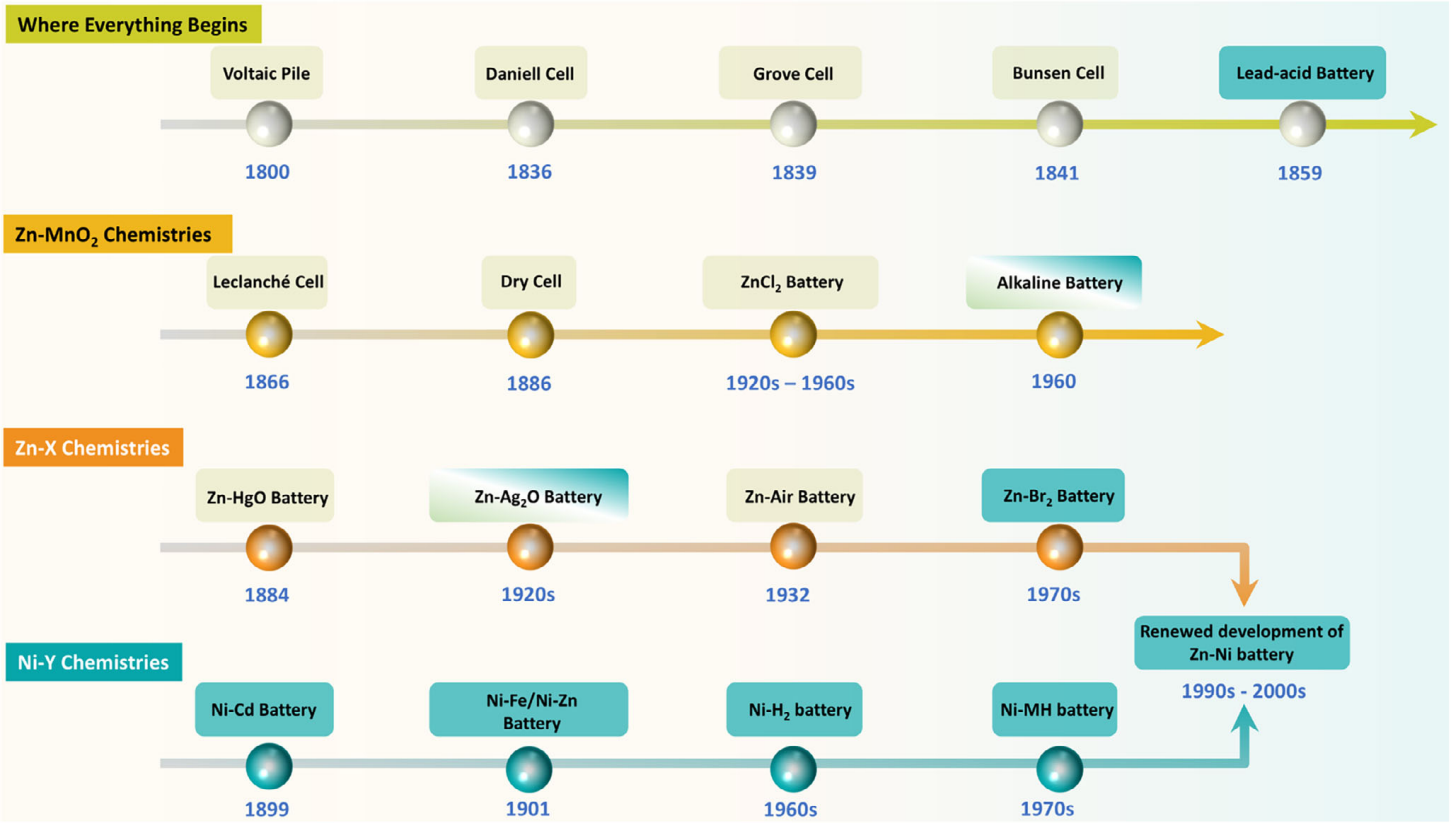
Historical timeline of major commercialized aqueous battery technologies.

Aqueous batteries, defined by their use of water‐based electrolytes, represent a pivotal phase in the evolution of electrochemical energy storage. Emerging in the 19th century and gaining prominence through the 20th, these systems powered early industrial, scientific, and consumer applications, setting the stage for modern battery technologies. As shown in Figure 2, this section traces their origins and early successes. We focus on key chemistries, such as zinc‐manganese dioxide (Zn‐MnO_2_), other Zn‐based chemistries with alternative cathodes (Zn‐X), and Ni‐based systems with varied anodes (Ni‐Y), highlighting their shared advantages of safety, cost, and environmental friendliness.

### 2.1 Where Everything Begins

**FIGURE 3:**
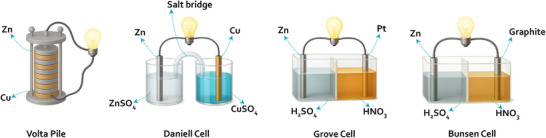
The Schemes of Representative Electrochemical Cells Invented in the 19th Century. The Volta pile established the first practical galvanic system through a stack of alternating Zn and Cu discs separated by cloth soaked in saline solution. The Daniell cell introduced chemical stabilization by pairing a Zn anode in ZnSO_4_ with a Cu cathode in CuSO_4_, using a porous barrier that maintained ionic conduction while suppressing hydrogen generation. The Grove cell increased output voltage by combining a Zn anode in diluted H_2_SO_4_ with a Pt cathode in concentrated HNO_3_, where the strong oxidizing capability of nitric acid enabled a potential near 1.9 V. The Bunsen cell reduced material cost and improved practicality by retaining the Grove configuration while substituting graphite for Pt at the cathode.

The story of aqueous batteries begins with the birth of battery technology itself, credited to the invention of Alessandro Volta, the volta pile (Figure 3) [1]. This rudimentary device, often hailed as the first electrochemical battery, demonstrated the potential of harnessing chemical reactions for electricity. Later on, the advancements came with the Daniell cell [26], the Grove cell [27], and the Bunsen cell [28]. The Daniell cell minimized gas buildup by ensuring that Cu^2+^ was reduced at the cathode rather than water (hydrogen evolution reaction), producing solid Cu instead of hydrogen in volta pile, while Grove cell and Bunsen cell yielded a higher voltage of approximately 1.9 V, thanks to the strong oxidizing power of nitric acid, which efficiently reduced at the cathode (Figure 3). These intermediate developments bridged the conceptual leap from the Volta pile to the first rechargeable aqueous system. By the mid‐19th century, these ideas culminated in the lead‐acid battery invented by Gaston Planté in 1859 [5]. Lead‐acid battery featuring Pb anodes and PbO_2_ cathodes in a H_2_SO_4_ electrolyte, marked a turning point. With a nominal voltage of 2 V, higher than earlier cells due to the Pb/PbO_2_ redox couple, and a robust, rechargeable design. Unlike its primary‐cell predecessors, the lead‐acid battery could be cycled by reversing the reaction (PbSO_4_ formation and dissolution), offering unprecedented utility. Its success cemented aqueous batteries as a cornerstone of industrial progress, proving that water‐based electrolytes could support both high voltage and reversibility. This pioneering era, from the Volta pile to the lead‐acid battery, underscored the versatility of aqueous systems, setting the stage for the diverse chemistries, Zn‐based, Ni‐based, and beyond, that would follow in the quest for reliable energy storage.

### 2.2 Zn‐MnO_2_ Chemistries

Among the earliest and most enduring aqueous battery technologies is the Zn‐MnO_2_ system, which emerged in the 19th century and evolved into a commercial mainstay by the mid‐20th century. This chemistry, rooted in simplicity and leveraging abundant materials, powered a range of applications from telegraphs to modern household devices. Its development spans several key iterations, the Leclanché cell, dry cell, ZnCl_2_ cell, and alkaline battery, each building on its predecessors to enhance performance, practicality, and market relevance.

**FIGURE 4:**
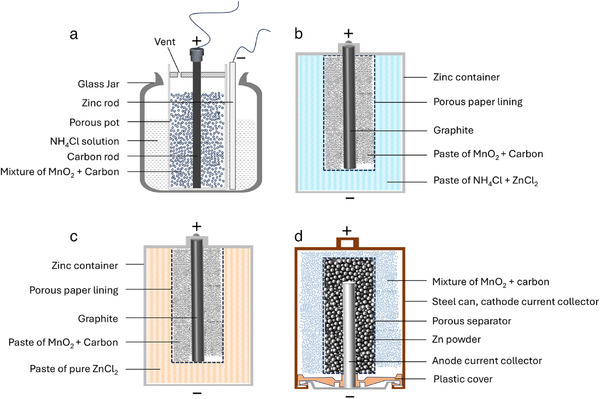
Structural evolution of Zn–MnO_2_ batteries from the 19th century to the modern alkaline cell. (a) Leclanché cell: a Zn metal anode and MnO_2_–carbon cathode immersed in NH_4_Cl aqueous electrolyte; simplicity and low cost defined this early design. (b) Dry cell: replaced the liquid electrolyte with a moist NH_4_Cl paste, encased in a sealed zinc can, improving portability and shelf life. (c) ZnCl_2_ cell: adopted ZnCl_2_ electrolyte to reduce corrosion and extend storage stability, while retaining the dry cell structure. (d) Alkaline battery: introduced KOH as the electrolyte and reversed the electrode configuration, with powdered zinc surrounding a central MnO_2_ cathode, greatly enhancing energy density and high‐drain performance.

The Leclanché cell [29, 30] is the first practical Zn‐MnO_2_ battery. As shown in Figure 4a, the Zn metal served as both the anode and current collector, while the MnO_2_ mixed with carbon to improve conductivity, was placed in a glass jar immersed in the electrolyte. Its liquid electrolyte posed leakage risks, and ammonia gas buildup reduced shelf life, limiting its portability and longevity. To address these drawbacks, Gassner replaced the liquid NH_4_Cl with a moist paste thickened with plaster of Paris or starch, encasing it in a Zn canister with a carbon rod inserted into the MnO_2_ cathode mixture [31]. This “dry cell” configuration (Figure 4b), though still aqueous in its electrolyte chemistry, mitigated leakage, making it portable and durable for consumer use. A parallel development emerged in the early 20th century with the ZnCl_2_ cell (Figure 4c) [32, 33, 34], a variant aimed at improving upon the performance of the dry cell. The ZnCl_2_ cell combines/replaces NH_4_Cl with ZnCl_2_ as the electrolyte to further reduce corrosion of the Zn anode, yielding less gaseous byproduct than the Leclanché cell. This iteration demonstrated the adaptability of Zn‐MnO_2_ chemistry to meet evolving needs, bridging early designs to more advanced systems. The pinnacle of Zn‐MnO_2_ evolution came with the alkaline battery, patented by Lewis Urry for Union Carbide in 1960 [35]. Replacing NH_4_Cl or ZnCl_2_ with a potassium hydroxide (KOH) electrolyte, the alkaline cell retained the zinc anode and MnO_2_ cathode but reversed the electrode configuration: powdered zinc was packed inside a perforated steel can, surrounded by a MnO_2_‐carbon mixture (Figure 4d). The cell maintained a similar output voltage but boosted energy density and power output significantly. Though still primary, their durability and efficiency solidified the legacy of Zn‐MnO_2_, with recent research exploring rechargeable variants using Cu‐intercalated MnO_2_, achieving over 6000 cycles in lab settings. A large prismatic rechargeable cell delivering ∼140 Wh/L is also demonstrated [36]. It is worth mentioning that the company Urban Electric Power, founded by the authors, is dedicated to developing rechargeable alkaline Zn–MnO_2_ batteries for large‐scale energy storage based on the above technology.

The progression from the Leclanché cell to the alkaline battery illustrates the enduring appeal of Zn‐MnO_2_ chemistries in aqueous battery technology. Each iteration addressed practical limitations, leakage, shelf life, capacity while retaining the core advantages of low cost and material abundance. These developments not only powered early technological advancements but also set the stage for modern efforts to revive Zn‐MnO_2_ chemistries for sustainable energy storage, reflecting their lasting impact on the field.

### 2.3 Zn‐X Chemistries (X: Different Cathodes Other Than MnO_2_)

Parallel to the development of Zn‐MnO_2_ systems, Zn‐based batteries with alternative cathodes (Zn‐X) emerged as researchers pursued higher energy density and rechargeability from the mid‐20th century onward. These chemistries, including Zn‐mercury oxide (Zn‐HgO), Zn‐silver oxide, Zn‐Air, Zn‐bromine (Zn‐Br_2_), and Zn‐Ni, pushed the boundaries of aqueous battery technology. Each offered unique advantages while facing challenges such as Zn dendrite growth, anode shape change, cathode instability, and environmental or cost‐related drawbacks.

The Zn‐HgO battery [37, 38, 39] marked a significant leap in compact, high‐density power sources, achieving a stable 1.35 V and up to 110 Wh/kg, double that of early Zn‐MnO_2_ cells [40]. The use of a mercury amalgam anode suppressed hydrogen evolution [41], enhancing stability over hundreds of discharge cycles. However, the toxicity of mercury became a big issue as environmental awareness rose in the 1970s [42]. Following Zn‐HgO, the Zn‐silver oxide battery [43, 44, 45, 46, 47] emerged in the 1960s. The primary reaction with Ag_2_O delivered 1.6 V and an energy density of up to 135 Wh/kg. This made Zn‐Ag_2_O ideal for aerospace (e.g., powering Apollo mission backup systems), medical devices (e.g., early pacemakers), and military uses (e.g., missile guidance) [48]. The high electronic conductivity of silver and the high ionic conductivity of the KOH electrolyte enabled exceptional power delivery, often exceeding 500 W/kg in short bursts [49, 50]. It is worth noting that in the year of 1966, Electrovair (a series of experimental EVs developed by General Motors) used rechargeable Zn‐silver oxide batteries and delivered roughly 40–80 miles per charge [51, 52]. In recent years, rechargeable Zn‐Silver oxide batteries have advanced significantly, driven by both academic and industrial efforts [53, 54, 55]. However, the high cost of silver restricted the Zn‐Silver oxide battery to niche markets. The trade‐off between energy density and economics highlighted potential and pitfalls of Zn‐based chemistries, inspiring later high‐energy but low‐cost designs like Zn‐Air and modern Zn‐ion systems. The Zn‐Air battery, with roots in 19th‐century experiments conducted by Leclanché [56], leveraging oxygen as a cathode reactant for maximum energy density [57]. Its open design, using ambient oxygen, gave a theoretical energy density over 1000 Wh/kg (including oxygen mass) [58, 59, 60]. Commercial Zn–Air cells were produced in the 1930s by firms such as National Carbon (Union Carbide) [61]. Miniaturized button‐type Zn–air cells for hearing aids did not arrive until the 1970s (commercialized in 1977) [62, 63]. Rechargeable Zn‐Air variants emerged late in the century, offering 100–200 cycles in labs, but dendrite formation and electrolyte drying hindered progress [64, 65]. The pursuit of ultra‐high energy density of Zn‐Air battery inspired other metal‐air systems (e.g., Fe‐Air, Li‐Air) [66, 67, 68], influencing modern research into overcoming its practical constraints for high energy density applications. The Zn‐Br_2_ battery, developed in the 1970s, introduced a flow‐battery chemistry, pairing a Zn anode with a Br_2_ cathode in a ZnBr_2_ aqueous electrolyte [69, 70, 71]. Pioneered by Exxon and Gould Inc., Zn‐Br_2_ targeted early grid storage, with prototypes powering small renewable installations by the 1980s [70]. Its flow design mitigated some dendrite issues by plating Zn metal on a static electrode, offering cycle lives of 500–1000 in controlled settings. However, the corrosiveness of Br_2_ required costly materials (e.g., Teflon), and complex plumbing limited commercial adoption [70, 72]. The scalability of Zn‐Br_2_ battery foreshadowed modern flow batteries like vanadium redox systems [73]. In the 1970s – 1980s, Zn–Ni batteries (Zn anode with NiOOH cathode in alkaline electrolyte) were extensively investigated as candidates for rechargeable aqueous systems. Typical cell voltages were around 1.6 V, and the energy densities approaching 120 Wh/kg [74, 75]. Companies such as PowerGenix Systems Inc. (now ZincFive) explored Zn–Ni technology for electric vehicles during the 1980s [76]. It is worth noting that in the early 21st century, Zn–Ni batteries, owing to their excellent high‐rate performance, found niche applications in power tools and other high‐drain, short‐duration devices [64]. More recently, driven by the rapid expansion of renewable energy, AI, and data infrastructure, ZincFive Inc. has introduced a series of high‐rate Zn‐Ni battery systems, boasting discharge capabilities exceeding 10 C, deployed as uninterruptible power supply (UPS) solutions in data centers [74, 77].

Collectively, Zn–HgO, Zn–Ag_2_O, Zn–Air, Zn–Br_2_, and Zn–Ni broadened the technological landscape of Zn‐based aqueous batteries. Despite their respective limitations, their development laid essential foundations for modern Zn‑ion and hybrid aqueous systems and demonstrated the versatility of Zn‐based chemistries across diverse performance regimes.

### 2.4 Ni‐Y Chemistries (Y: Different Anodes)

As illustrated in Figure 2, Zn‐X and Ni‐Y chemistries evolved in parallel from the mid‐20th century onward. The former broadened Zn‐based options through diverse cathodes, while the latter was built around Ni(OH)_2_/NiOOH cathodes paired with different anodes. Rather than separate tracks, the two families converged at the Zn–Ni battery. It is worth noting that although Zn‐X chemistries included some rechargeable attempts, they were primarily developed as primary batteries with a focus on gradually increasing energy density. By contrast, Ni‐Y systems were essentially conceived as rechargeable chemistries, achieving substantial improvements in cycle life and practical rechargeability. Building on this intersection, the following section turns to the development of Ni‐Y systems.

Ni‐based aqueous batteries, featuring Ni(OH)_2_ or NiOOH cathodes paired with various anodes (Ni‐Y), emerged as foundational rechargeable systems from the late 19th to 20th century [78, 79]. Using KOH electrolytes, these Ni‐Y batteries (including Ni‐Cd, Ni‐Fe, Ni‐H_2_, Ni‐MH, and Ni‐Zn) capitalized on the reversibility and stability of NiOOH/Ni(OH)_2_ redox, offering durability and versatility. Among the earliest Ni‐Y systems, the Ni‐Cd battery invented by Waldemar Jungner in 1899, paired a NiOOH cathode with a Cd anode in KOH, delivering 1.2 V and 40–60 Wh/kg [80]. Known for robustness, Ni‐Cd tolerated overcharging and extreme temperatures (−50°C to 60°C) [81, 82], with cycle life often reaching into the thousands (e.g. ∼1000 cycles in aerospace settings) [83, 84, 85], However, the intrinsic toxicity of Cd and the well‐documented memory effect gradually undermined its competitiveness of [86]. Soon after, the Ni‐Fe battery, invented by Thomas Edison and introduced in 1901, provided an alternative Ni‐Y chemistry distinguished by exceptional durability [87], with some cells operational after 100+ years, making it a favored choice for harsh and continuous‐duty applications [88]. Decades later, the Ni‐H_2_ battery, developed in the 1970s by COMSAT and Intelsat, extended the Ni‐Y family into aerospace applications [89, 90, 91]. Pairing a NiOOH cathode with a H_2_ anode in a pressurized vessel (50–100 atm), a Ni‐H_2_ battery delivered 1.2–1.3 V and 50–75 Wh/kg [89, 90, 91]. Ni‐H2‐powered satellites like the Hubble Space Telescope and International Space Station, excelling in zero‐gravity with near‐zero self‐discharge (0.001% daily). While classic Ni‐H_2_ systems have historically been constrained by the cost of platinum catalysts, pressurized vessels, and system complexity, recent innovations aim to overcome these barriers. For instance, EnerVenue currently promotes Ni‐H_2_ battery technology designed to exceed 30 000 cycles and leverages lower‐cost alloy catalysts to reduce reliance on platinum [92, 93, 94, 95]. In parallel with aerospace developments, Matsushita and Sanyo commercialized Ni‐MH batteries in the 1980s, which replaced H_2_ gas electrode with hydrogen‐absorbing alloys (e.g., LaNi_5_ or AB_5_‐type alloys) [96]. Modern Ni‐MH cells typically operate at ∼1.2 V, and deliver energy density of 60–120 Wh/kg. Ni‐MH powered early EVs, such as the Toyota RAV4 (1996) and Toyota Prius (1997). Self‐discharge (20%–30% monthly) remained a challenge, but Ni‐MH bridged aqueous systems to modern hybrid technology before lithium‐ion dominance. The Ni‐Zn (or Zn‐Ni as mentioned above) battery, which bridges the Zn‐X and Ni‐Y categories, emerged in the 1970s. Ni–Zn batteries took advantage of higher cell voltage compared to other Ni‐Y systems by pairing zinc anodes with NiOOH cathodes.

By leveraging the robust redox chemistry of Ni(OH)_2_/NiOOH, Ni‐Y batteries provided durable and versatile rechargeable platforms that profoundly influenced the evolution of modern electrochemical energy storage.

### 2.5 Performance Overview of Historical Aqueous Battery Chemistries

**FIGURE 5:**
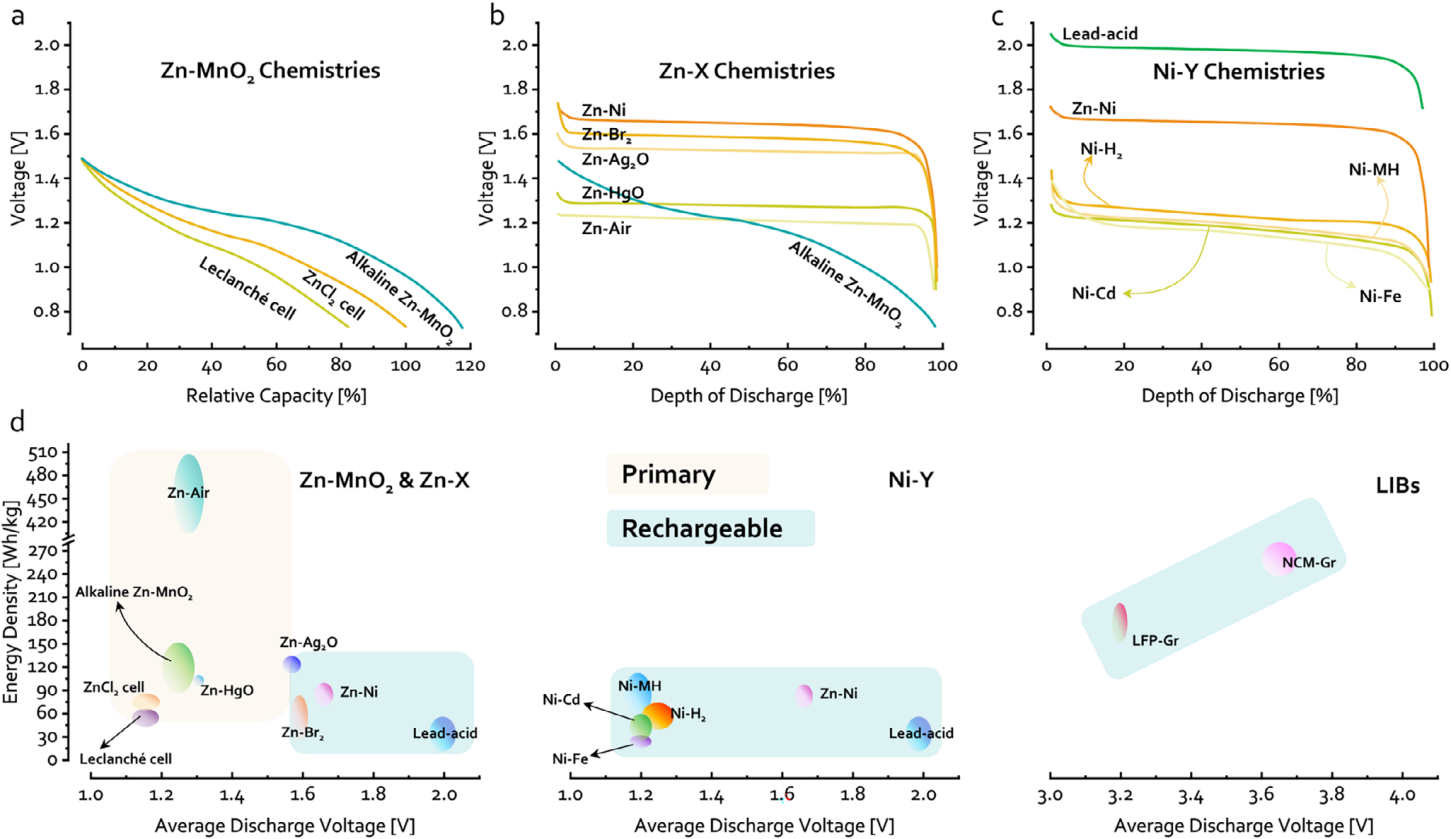
Typical discharge curves of the (a) Zn‐MnO_2_ chemistries, (b) Zn‐X Chemistries, and (c) Ni‐Y Chemistries. (d) The voltage and energy density plots of different batteries, LFP‐Gr and NCM‐Gr, are also plotted for reference [64]. All battery energy density values refer to the cell‐level performance.

In Figure 5a–c, we compile representative discharge curves for the aqueous batteries discussed above. For Zn‐MnO_2_ chemistries, progressive optimization of the electrolyte from NH_4_Cl to KOH leads to a continuous increase in accessible discharge capacity, and therefore energy density (Figure 5d). This development underscores the pivotal role of electrolyte design in battery development. As the chemistry evolves from Zn‐MnO_2_ to other Zn‐X systems, the discharge voltage plateau systematically increases, following the order NiOOH > Br_2_> Ag_2_O > MnO_2_. Although Zn‐Air operates at the lowest voltage, their exceptionally high specific capacity yields the highest overall energy density among these chemistries. This historical evolution highlights two direct and intuitive routes to enhancing battery energy density: increasing specific capacity or increasing cell voltage. To further achieve the transition from primary to secondary aqueous batteries, researchers centered on NiOOH cathodes and systematically explored alternative anode materials, thereby developing a series of rechargeable systems with progressively improved energy density and cycle life.

Figure 5d further summarizes the operating voltage and gravimetric energy density of these batteries. In general, primary batteries exhibit higher energy densities than their rechargeable counterparts. Overall, the majority of systems deliver energy densities below 100 Wh/kg. Even the costly Zn‐Ag_2_O chemistry remains below 150 Wh/kg. These values fall far short of those of modern LIBs and thus offer limited competitiveness for current high‐energy applications.

### 2.6 Key Advantages: Safety, Cost, and Environmental Benefits

The early success of aqueous batteries rested on three core advantages that distinguished them from later non‐aqueous systems.

Safety: Water‐based electrolytes are inherently non‐flammable, unlike the organic solvents in LIBs, minimizing fire and explosion risks [25]. This attribute was decisive in confined or rugged environments. For instance, lead‐acid batteries in submarines or Ni–Cd packs in military radios and aviation backup systems, where failure tolerance was essential [97, 98].

**FIGURE 6:**
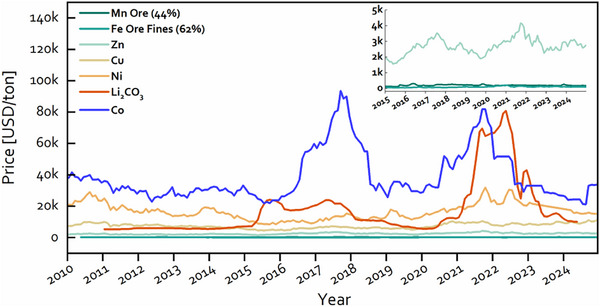
Historical prices of different metals. Data from Investing.com and Trading Economics.

Cost: The reliance on abundant, inexpensive raw materials such as lead, zinc, manganese, and nickel (Figure 6) enabled large‐scale deployment at relatively low prices (noting that Ni can be more expensive and price‐volatile than Fe or Mn, yet it typically remains less cost‐ and supply‐constrained than Li‐ or Co‐dependent materials). Lead‐acid batteries became the archetype of low‐cost mass production, powering both industrial equipment and early automobiles, while Zn–MnO_2_ dry cells were cheap enough to transform consumer electronics into everyday commodities.

Environmental considerations: Compared with later technologies that depend on scarce and toxic elements (e.g., cobalt in LIBs), many aqueous chemistries made use of metals that were more recyclable and less environmentally hazardous. The shift from cadmium to hydrogen‐storage alloys in Ni–MH exemplified the ongoing effort to reduce toxicity and align with growing environmental awareness, even before regulatory frameworks fully emerged. Moreover, recycling aqueous batteries is generally more economical and environmentally friendly, as their water‐based electrolytes and relatively benign components allow for simpler separation processes and lower energy inputs. In contrast, recycling LIBs often requires complex, high‐temperature or solvent‐intensive treatments, which not only pose safety risks but also lead to higher carbon emissions and chemical waste [99, 100]. This fundamental difference underscored how aqueous systems were, in many respects, better aligned with circular economy principles long before sustainability became a mainstream priority.

Taken together, these strengths explain why aqueous batteries dominated electrochemical energy storage from the Victorian era through the mid‐20th century. Their combination of safety, affordability, and relative environmental compatibility allowed them to penetrate both heavy‐duty industrial applications and mass consumer markets. However, these very advantages also masked underlying weaknesses. While aqueous batteries excelled in safety and cost‐effectiveness, they were ultimately constrained by limited electrochemical windows, structural degradation mechanisms, and performance ceilings that became increasingly evident as the demand for portable, high‐energy storage intensified in the late 20th century. The next section examines how these technical and market forces combined to bring about their decline, paving the way for the rise of lithium‐ion technology.

## 3 The Decline: Why Aqueous Batteries Lost to Lithium‐Ion

The initial success and widespread adoption of aqueous batteries in diverse applications were eventually overshadowed by intrinsic limitations that paved the way for the dominance of LIBs. While aqueous systems offered inherent safety, affordability, and environmental advantages, their shortcomings in energy density, cycling stability, and adaptability to emerging portable and high‐energy applications ultimately led to their decline.

### 3.1 Energy Density Limitations and Electrochemical Stability

A primary constraint of aqueous batteries lay in their limited energy density, a decisive metric for modern electronics and electric vehicles. The electrochemical stability window of water (∼1.23 V) fundamentally restricted the achievable voltage, as water decomposes into hydrogen and oxygen beyond this limit [25]. As a result, even the best aqueous systems lagged far behind non‐aqueous alternatives. The ceiling of energy density of the rechargeable aqueous batteries is about 120 Wh/kg. In stark contrast, LIBs offered cell voltages exceeding 3.7 V and practical energy densities of 250–300 Wh/kg, with lithium‐metal variants today surpassing 400–600 Wh/kg [101, 102, 103]. This widening gap made LIBs far more attractive for compact, high‐power applications.

The narrow electrochemical stability window also constrained the choice of electrode materials in aqueous systems, as many candidates triggered parasitic water electrolysis. By contrast, non‐aqueous electrolytes enabled LIBs to exploit a much broader voltage range and a richer palette of electrode chemistries, providing greater flexibility for optimization.

### 3.2 Structural Degradation and Poor Cycling Performance

Beyond voltage and energy density, aqueous batteries were hindered by structural degradation and poor cycling stability [25, 104]. Most electrode reactions in aqueous systems involve conversion or phase‐change mechanisms, leading to large volume variations and crystallographic rearrangements during cycling [98]. These transformations often induced cracking, pulverization, and contact loss, undermining long‐term stability. Classic examples illustrate these limitations. In lead–acid batteries, the repeated formation and dissolution of PbSO_4_ crystals, especially coarse, stable deposits (sulfation) reduced active material utilization and shortened cycle life [105, 106]. Similarly, zinc‐based batteries relied on zinc dissolution and redeposition; repeated cycles promoted uneven deposition and dendritic growth, which not only reduced Coulombic efficiency but also risked short circuits and catastrophic failure [107, 108, 109]. By contrast, LIB electrodes primarily operate through intercalation, where lithium ions reversibly insert into host lattices with minimal structural disruption. Examples include widely used cathode materials like lithium cobalt oxide (LCO), nickel‐cobalt‐manganese (NCM), lithium iron phosphate (LFP), and lithium manganese oxide (LMO), as well as anode materials like lithium titanate (LTO) and graphite (Gr). This intercalation mechanism allows for much better structural integrity and cycling stability over thousands of cycles, a key factor of superior performance and longevity of LIBs [110, 111].

Even nickel‐based aqueous chemistries, once considered robust, revealed intrinsic drawbacks. Ni–Cd batteries could reach 1000–2000 cycles but were plagued by the well‐known “memory effect,” [7] while Ni–MH cells overcame cadmium toxicity yet suffered from severe self‐discharge (20%–30% monthly). These degradation pathways limited aqueous systems to meet the growing demand for long‐lasting, high‐performance rechargeable storage.

### 3.3 Market Forces and the Rise of LIBs

Technical limitations alone did not seal the fate of aqueous batteries; market forces accelerated their decline. From the 1980s onward, the consumer electronics revolution drove demand for lightweight, compact, and long‐lasting energy storage in laptops, camcorders, and later smartphones. LIBs, with their unmatched energy density and cycle stability, aligned perfectly with these requirements and rapidly became the industry standard.

The rise of electric vehicles and the emerging need for grid‐scale storage further reinforced LIB dominance. Massive investments in R&D, manufacturing capacity, and supply chains created economies of scale that continually improved LIB performance while reducing cost. This virtuous cycle of innovation and commercialization further widened the gap with aqueous systems. As a result, they became confined to niche roles such as automotive starter batteries, backup power supplies, and low‐power applications, where safety and affordability remained advantageous.

## 4 The Revival: Recent Innovations and Technological Breakthroughs

**FIGURE 7:**
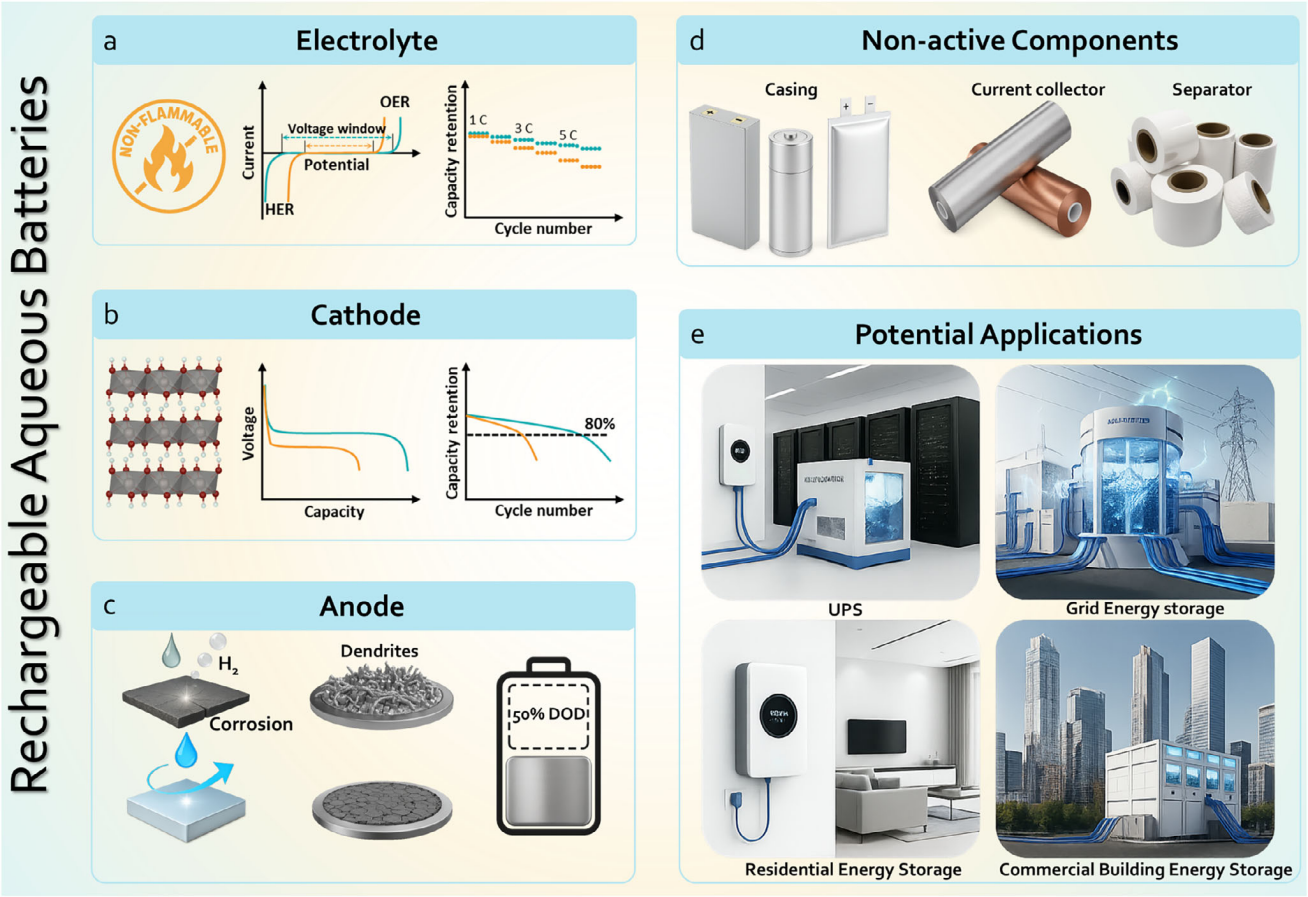
Recent Advancements and Emerging Directions in Aqueous Batteries. (a) Electrolyte design plays a critical role in enabling the non‐flammable nature of aqueous systems. Strategies to expand the voltage window, improve rate capability, and enhance cycling stability are essential. (b) Cathode materials largely determine the energy density of aqueous batteries. Desirable features include high specific capacity, elevated average discharge voltage, and long‐term cycling durability. (c) Zn anodes, despite over two centuries of use, continue to face persistent challenges such as corrosion, hydrogen evolution, and dendrite growth, while improving Zn utilization remains crucial for boosting energy density. (d) Non‐active components (e.g., casings, current collectors, separators) also significantly influence battery cost, gravimetric/volumetric energy density, and long‐term stability, and thus require thoughtful engineering. (e) Potential applications for aqueous batteries include residential and commercial energy storage, UPS, and large‐scale grid storage, where safety, cost‐effectiveness, and sustainability are paramount.

Aqueous batteries have experienced a remarkable resurgence over the past decade. Driven by the growing demand for sustainable, safe, and cost‐effective energy storage solutions. Recent advancements in electrolyte design, electrode materials, and application‐oriented developments have significantly enhanced their performance. These breakthroughs, particularly in improving energy density (via higher operating voltages and specific capacities of the electrode materials) and extending cycle life, have unlocked immense potential for grid‐scale energy storage and specialized applications (Figure 7).

The primary bottlenecks of aqueous batteries, namely the energy density (determined by operating voltage and electrode specific capacity) and lifespan, have been the focal points of recent innovations. Advances in electrolyte engineering, novel electrode materials that are devoted to these challenges, as detailed below.

### 4.1 Innovative Electrolyte Design

Innovative electrolyte designs have been critical in overcoming the inherent electrochemical stability window (ESW) limitations of water, enhancing the cyclability of the electrode materials, thus enabling higher operating voltages and enhanced cycling performance (Figure 7a).

#### 4.1.1 Water‐in‐Salt Electrolytes

The “Water‐in‐Salt” Electrolyte (WiSE), a groundbreaking development, involves dissolving a very high concentration of salt, such as 21 mol/kg LiTFSI, in a minimal amount of water [24]. This dramatically alters the solvation structure of ions, reducing the activity of “free water” and forming a robust LiF‐rich solid‐electrolyte interphase (SEI) on anodes. Consequently, WiSE effectively suppresses parasitic reactions like hydrogen evolution (HER) and oxygen evolution (OER), significantly extending the ESW to ∼3.0 V, allowing for higher voltage operation and improved energy density. Innovations like hydrate‐melt electrolytes further widened the ESW to 3.8 V [112], while “water‐in‐bisalt” accompanied with a fluorinated additive supporting high‐capacity anodes like Gr or lithium metal [113]. Despite challenges such as high cost and viscosity, recent advancements enhance SEI stability and ionic conductivity, achieving stable cycling over 1000 cycles. The versatility of WiSE extends to sodium, zinc, and magnesium batteries [114, 115], bridging the energy density gap with non‐aqueous systems while offering safer, greener energy storage solutions.

#### 4.1.2 Decoupled Electrolyte Design

Following the rise of WiSE strategies, pH‐decoupled electrolytes (i.e., employing different pH media for cathode and anode) have emerged as an alternative route to push aqueous battery voltages beyond 1.23 V limit. The key idea is to spatially separate the positive and negative electrodes in acidic and alkaline electrolytes, respectively, with an ion‐selective membrane in between, thus decoupling the redox environments at each electrode. This allows each half‐cell to operate nearer its stable potential window and enables full cells to reach voltages above 2.5–3.0 V while still maintaining aqueous safety. As an example, Zhong et al. [116]. demonstrated a 2.83 V Zn‐MnO_2_ aqueous battery. In their design, the cathode operates in an acidic medium (MnO_2_/Mn^2+^ chemistry), while the anode uses Zn/Zn(OH)_4_^2−^ in alkaline media. Moreover, in the same realm of decoupled designs, Zhu et al. discuss the broader category of pH‐decoupled aqueous batteries, summarizing prototypes where acidic catholytes are combined with alkaline anolytes to broaden the operational voltage window above 3 V, enabled by selective membranes and tuned redox couples [117]. Another type of decoupled design is an aqueous/organic hybrid electrolyte. Niu et al. designed such an electrolyte for Li‐S battery which achieved a high discharge voltage of 3.4 V and high capacity of 2300 mAh g^−1^ [118].

While these strategies are still in relatively early stages, they highlight a promising pathway to circumvent the classical water voltage limitation without abandoning the safety advantages of aqueous systems.

#### 4.1.3 Water/Organic Solvent Hybrid Electrolyte

Another promising approach to overcome the intrinsic electrochemical stability window of water is the use of water/organic solvent hybrid electrolytes. By partially replacing water with miscible or even immiscible organic solvents, such as acetonitrile, ethylene carbonate, dimethyl sulfoxide, or glymes, the hydrogen‐bonding network of water is disrupted and the overall solvation structure of cations is significantly modified [119, 120, 121, 122]. This dual‐solvent environment reduces the activity of free water molecules, thereby suppressing parasitic side reactions like hydrogen evolution while widening the accessible voltage window. Moreover, the introduction of organic cosolvents improves interfacial compatibility with both positive and negative electrodes, enabling reversible cycling of materials that are unstable in purely aqueous environments. For example, hybrid electrolytes have been shown to stabilize zinc anodes by mitigating dendrite formation and corrosion [120]. Compared with fully organic systems, water/organic hybrids retain the intrinsic nonflammability and ionic conductivity benefits of water, while the organic fraction provides extended stability and flexibility in electrolyte design. Despite challenges such as volatility, flammability of certain solvents, and long‐term chemical stability, recent studies demonstrate that optimized hybrid formulations can achieve cell voltages approaching 3 V with improved Coulombic efficiency and cycle life. As a result, water/organic hybrid electrolytes represent a versatile and scalable pathway to balance safety, energy density, and cost in next‐generation aqueous batteries.

Overall, these innovative electrolyte strategies, including water‐in‐salt, pH‐decoupled, and water/organic hybrid formulations have each proven highly effective in expanding the voltage window and enhancing the cycling stability of aqueous batteries. Nevertheless, all approaches face their own limitations, whether in cost, complexity, kinetics, or long‐term stability. Besides, these modern aqueous electrolytes often compromise the inherent sustainability advantage of aqueous systems due to the use of organic salts and solvents. Collectively, these strategies highlight the central role of electrolyte design in redefining the electrochemical window of water, but also underscore the need for continued innovation to reconcile performance gains with cost, safety, and sustainability.

### 4.2 Advanced Electrode Materials

Following the breakthroughs in electrolyte innovations, the development of advanced electrode materials is crucial for addressing the energy density and cycle life limitations of aqueous batteries, with significant progress in anode and cathode materials (Figure 7b).

#### 4.2.1 Anode Materials

Many anodes in aqueous systems have been explored recently [123, 124, 125]. Among them, Sn‐based aqueous batteries have recently undergone a notable paradigm shift from acidic to alkaline ones, mirroring the historical trajectory of Zn‐based chemistries. In 2023, Chao et al. demonstrated a highly reversible alkaline Sn metal anode enabled by stannite‐ion electrochemistry (−1.0 V vs. SHE), which extends the accessible negative potential window and enables markedly higher operating voltages of Sn‐based full cells, compared to conventional acidic Sn^2^^+^/Sn systems (−0.13 V vs. SHE) [126, 127]. Despite these advances that broaden the aqueous anode landscape, Zn remains the most extensively studied and practically relevant anode for rechargeable aqueous batteries, owing to its compelling combination of properties [128, 129, 130]. The study of Zn anodes has a long and significant history, with the first research efforts dating back to 1800 [1]. As also depicted in Figure 1, zinc has recently seen a resurgence in interest and has become a primary research focus. Zn anodes are favored in aqueous batteries for abundance, low cost, suitable redox potential (∼ −0.76 V vs Standard Hydrogen Electrode, SHE), and high theoretical capacities (∼820 mAh g^−1^; ∼5850 mAh cm^−3^), which enable compact, safe storage [120, 131, 132, 133, 134]. Yet practical Zn anodes still contend with several intertwined failure modes: dendritic growth, corrosion/passivation, shape change, and HER, all of which lower Coulombic efficiency and shorten life. Besides traditional approaches, recent years have seen the emergence of new methods [135, 136, 137], which primarily focus on Zn surface coating, SEI design, and electrolyte regulation [130, 138, 139, 140, 141, 142, 143, 144]. Notably, Archer et al. developed a graphene coated stainless‐steel electrode, which guided uniform Zn deposition with preferential orientation parallel to the electrode due to the low lattice mismatch between graphene and metallic zinc. This epitaxial Zn anode enables highly reversible cycling with ∼99% Coulombic efficiency maintained over 1000 cycles [138]. Chen et al. designed a robust bilayer SEI on Zn anodes by introducing 1,3‐dimethyl‐2‐imidazolidinone as an electrolyte additive. The bilayer comprising a ZnCO_3_‐rich crystalline outer layer and a ZnS‐rich amorphous inner layer, significantly enhanced Coulombic efficiency (∼99.95%) and cycling stability (4800 cycles), enabling uniform Zn deposition and high Zn utilization [142].

While there have been numerous advancements in protecting zinc anodes, we must pay closer attention to the real‐world performance of these strategies under practical and commercial conditions. Key considerations include high depth of discharge (e.g., 50% DOD) which reflects the utilization ratio of the Zn metal anode, or in other words, the negative to positive electrode capacity ratio (N/P ratio), high areal capacity (3–5 mAh/cm^2^), electrolyte to capacity ratio (E/C ratio in the unit of g/Ah), self‐discharge, and shelf/calendar life evaluation in Ah‐level cells. To enhance comparability and avoid overly optimistic projections from coin‐cell results to pouch‐scale systems, it is crucial to report these practical metrics. Detailed discussions of these factors can be found in previously published review papers [145, 146, 147, 148].

#### 4.2.2 Cathode Materials

Developing high‐performance cathodes remains a central challenge in advancing aqueous batteries (see co‐occurrence network in Figure 1), as their energy density and cycle life hinge on overcoming material‐specific limitations.

Vanadium‐based (V‐based), manganese‐based (Mn‐based), and Prussian blue analog (PBA) materials are primary candidates, each with distinct advantages and drawbacks [131]. V‐based cathodes, such as V_2_O_5_, offer multi‐electron redox capabilities but are limited by high costs, vanadium dissolution, and low voltages (∼0.8–1.0 V vs. Zn/Zn^2+^), constraining energy output and scalability [149, 150]. PBA cathodes provide excellent structural stability and fast ion diffusion, ideal for long‐term cycling, but their low specific capacities (∼50–70 mAh/g) limit their suitability for high‐energy applications [151, 152]. Mn‐based cathodes, particularly MnO_2_, are promising due to their low cost, natural abundance, and high theoretical capacity (∼308 mAh/g based on one‐electron redox) [153]. However, the structural and electrochemical stability issues are the main obstacles to overcome. Conventional intercalation‐type MnO_2_, such as α‐MnO_2_ with tunnel structures or δ‐MnO_2_ with layered structures, relies on Zn^2+^ or H^+^ insertion/extraction [154, 155]. A more transformative approach involves MnO_2_ cathodes leveraging dissolution‐deposition reactions. Chen et al. [156]. developed a rechargeable manganese–hydrogen battery that couples the reversible Mn^2+^/MnO^2^ redox reaction with a hydrogen evolution/oxidation electrode, achieving over 10 000 stable cycles. This mechanism surpasses intercalation by enabling deposition/dissolution processes, enhancing capacity and voltage through soluble Mn^2+^ intermediates that redeposit during charging. However, challenges include low Coulombic efficiency due to disproportionation reactions (e.g., 2Mn^3+^ → Mn^2+^ + Mn^4+^), accumulation of inactive “dead” MnO_2_ along with cycling, poor electrical conductivity. Besides, the Mn^2+^/MnO_2_ redox reaction relies on acidic electrolytes which is incompatible with Zn anodes, complicating full‐cell design.

Beyond conventional intercalation‐type cathodes, sulfur‐based cathodes have recently emerged as a conceptually distinct class of conversion‐type active materials for high‐capacity aqueous batteries [157]. In this context, D. Chao is a pioneer who revealed the unique interaction between H_2_O and S species after the Volmer step of water decomposition, which is fundamentally distinct from that in organic sulfur batteries [158]. In 2024, the group first proposed a high‐valent sulfur redox chemistry in aqueous sulfur batteries [159]. Overcoming the intrinsic voltage limitation associated with conventional low‐valent polysulfide redox (E_0_ = −0.51 V vs. SHE), the new redox features both high capacity and positive redox potential (E_0_> 0 V vs. SHE). Building on this conceptual advance, a thiosulfate‐mediated sulfur redox regulation strategy was further developed, enabling high capacity over 2000 mAh/g with high sulfur utilization [160].

Another typical conversion‐type cathodes are halogen materials. While Zn–Br_2_ batteries historically showcased the potential of halogen‐based systems, issues related to the corrosiveness and volatility of Br_2_ posed safety and engineering challenges. I_2_, as a less aggressive halogen, has drawn increasing attention as an alternative. However, the solid–liquid phase transition of I_2_ during cycling presents significant hurdles in achieving long‐term reversibility and material retention. Pan et al. [161]. demonstrated that encapsulation of iodine in microporous carbon hosts can stabilize reversible conversion of iodine species in water, enabling stable cyclic life over 3000 cycles with ∼90% capacity retention, and negligible self‐discharge. More importantly, recent work on interhalogen compounds, such as iodine monochloride (ICl) or iodine monobromide (IBr), offers further potential by stabilizing reactive intermediates and enhancing capacity [162, 163, 164, 165, 166]. For instance, Liang et al. demonstrated a four‑electron aqueous Zn–I_2_ battery by activating the highly reversible I_2_/I^+^ couple (1.83 V vs. Zn/Zn^2+^) in addition to the conventional I^−^/I_2_ couple (1.29 V), achieved through intensive electrolyte solvation that forms interhalogen ICl species and suppresses their hydrolysis [162]. More recently, Chen et al. expanded this concept by exploiting interhalogen chemistry to realize up to eight‐electron transfer redox reactions, achieving an impressive specific capacity of 809.2 mAh/g in a finely tuned “chloride‐in‐acid” electrolyte [165]. These breakthroughs underscore the significant potential of interhalogen systems for developing high‐energy‐density aqueous batteries. However, when leveraging the high capacity of these soluble halogen species, similar to Li‐S chemistries, the “shuttle effect,” including issues such as self‐discharge and anode/cathode crossover, must be carefully managed.

Besides, organic cathode materials have garnered growing interest due to their structural tunability, sustainability, and compatibility with aqueous systems. Their molecular diversity enables tailoring of redox potentials and solubility through rational design [167, 168, 169]. However, most organic cathodes suffer from poor cycle stability due to dissolution in aqueous electrolytes, sluggish electronic conductivity, and side reactions during redox cycling. In addition, their low density (∼1.2–1.5 g/cm^3^) compared to metal oxides leads to reduced volumetric energy density. How to overcome issues such as solubility, conductivity, and density remains a central focus in the future development of organic cathode materials for aqueous batteries.

Compared with anodes (with Zn currently the most promising candidate), cathode materials present a broader but less resolved landscape. No single class has yet established dominance, highlighting that while stabilizing Zn anodes remains essential, systematic exploration and targeted optimization of cathodes are equally critical. Cathodes play a decisive role in determining the energy density of aqueous batteries, as both cell voltage and capacity are largely governed by cathode chemistry. Their long‐term stability directly impacts cycle life, while raw material cost and abundance affect commercial scalability. Moreover, compatibility with aqueous electrolytes influences processability and design flexibility. Therefore, cathode innovation is not merely a supplement to anode engineering, but a central axis that will shape the practical viability and competitiveness of next‐generation aqueous battery systems.

### 4.3 Application‐Oriented Developments and Market Potential

Market demand is a powerful driver of technological innovation, often catalyzing breakthroughs to meet pressing societal and industrial needs. Historically, technologies like radar, GPS, and lithium‐ion batteries saw rapid development spurred by urgent demands, including those driven by wartime necessities. Similarly, the renewed interest in aqueous batteries is not a coincidence. It is a direct response to shifting market demands for safer, more sustainable, and cost‐effective energy storage solutions. While LIBs have dominated the past three decades, growing concerns over thermal safety, supply chain risks, and end‐of‐life recycling challenges have created space for alternative technologies better suited to certain applications. Aqueous batteries, once considered outdated, are now making a comeback by aligning naturally with these evolving needs. Besides the need for energy storage due to the intermittency of renewable energy, another critical driver is the rise of artificial intelligence (AI) infrastructure and large‐scale data centers, where the demand for robust, uninterruptible power supply (UPS) systems has surged. This market‐driven momentum is a key force propelling research and development in aqueous battery technologies. In this context, aqueous systems like Zn‐Ni batteries offer high‐rate discharge capability, inherent safety (non‐flammable electrolytes), and operational stability under a wide temperature range, making them strong candidates for next‐generation UPS systems. In parallel, the global transition toward renewable energy has amplified the need for long‐duration energy storage to buffer the intermittency of solar and wind. Fe‐air, Zn–MnO_2_, and Ni–H_2_ systems have shown increasing viability for this role, offering multi‐hour discharge capability, low materials cost, and simplified thermal management, with companies like Form Energy, Urban Electric Power, and EnerVenue pushing toward commercialization.

At the same time, regulatory and societal pressure for greener technologies has intensified. Aqueous batteries free from critical or toxic metals like cobalt or cadmium and offer simplified, low‐emission recycling pathways, are increasingly viewed as a more environmentally responsible choice for stationary applications [170].

Together, these application‐driven factors and sustainability imperatives are redefining the value proposition of aqueous batteries. No longer confined to historical footnotes, they are emerging as fit‐for‐purpose solutions in a rapidly diversifying energy storage landscape.

## 5 Future Perspectives: Can Aqueous Batteries Compete Again?

The future of aqueous batteries does not lie in directly competing with lithium‐ion batteries in applications demanding ultrahigh energy density (e.g., smartphones or long‐range EVs). Instead, their strength is in occupying complementary markets where intrinsic advantages, safety, low cost, and recyclability, are most valued.

### 5.1 Applications for Aqueous Batteries

Grid‐scale storage stands out as the most promising application domain, where the key requirements are long service life, low cost per kWh, and operational safety rather than compactness. In this context, aqueous batteries, particularly Zn‐based and Fe‐based chemistries are well‐positioned to meet these demands. However, it is essential to recognize that electrochemical performance must align with the actual needs of target applications.

First, grid systems typically operate under low current densities and require lifespans exceeding 15–20 years. In contrast, many aqueous batteries, such as Zn‐based systems, suffer from durability issues due to the thermodynamic instability of the Zn anode in aqueous solutions. While Zn‐based batteries have demonstrated thousands of stable cycles at high current densities in the lab, such high‐rate testing cannot be directly extrapolated to low‐rate operation. Fast cycling often suppresses side reactions, which may artificially suggest excellent stability. Moreover, cycle life testing does not fully capture the long‐term stability required for practical applications, particularly for grid‐scale energy storage. A key concern in aqueous systems is corrosion, which is a time‐dependent, parasitic reaction that occurs continuously, regardless of cycling. This phenomenon is not addressed by traditional cycle life testing, and a battery that survives 10 000 cycles in 3 months of fast charging may fail within a year on the shelf due to corrosion. Thus, calendar life, defined as resistance to time‐dependent degradation mechanisms such as corrosion becomes the true ‘missing link’ in current aqueous battery research. Unlike cycle life, which focuses on performance during cycling, calendar life addresses long‐term stability during storage and at rest, which is crucial for real‐world applications where batteries may not be in constant use. Additionally, there is still a lack of durability validation in practical, large‐format prototypes (e.g., 5–10 Ah pouch cells), which hinders commercial translation. For grid applications, it is essential to evaluate not just cycle life, but also calendar life, to ensure that the batteries can survive the 15–20‐year lifespan required in the field.

Another often‐overlooked barrier is system‐level integration cost. Due to the lower output voltage of aqueous cells (1.2–1.8 V vs. 3.2–3.7 V for LIBs), power converters (e.g., inverters, direct current (DC)–DC converters) require additional engineering to achieve voltage matching and efficiency. Meanwhile, they require higher currents to deliver the same power, which places greater demand on busbars and cabling (I^2^R losses). In addition, many aqueous chemistries (especially conversion‐type MnO_2_ or those involving severe voltage hysteresis) suffer from lower energy efficiency compared to Li‐ion (>90%). These factors could lead to higher balance‐of‐system costs compared to LIB‐based systems, which should be carefully considered in techno‐economic evaluations.

Beyond utility‐scale storage, aqueous batteries also offer value in decentralized or safety‐critical applications, such as residential solar systems, rural electrification, backup power for hospitals, telecom towers, and UPS for data centers. In dense urban environments, their non‐flammable water‐based electrolytes provide a compelling advantage, particularly amid growing fire concerns associated with LIBs. Further, aqueous chemistries are being explored in wearable medical devices, aerospace systems, and hybrid architectures that combine aqueous and non‐aqueous cells for tailored performance. In addition, given the high power and low‐cost characteristics of aqueous batteries, e‐bikes and automotive start‐stop power systems are also potential application areas for aqueous batteries.

### 5.2 Policy and Regulatory Frameworks

The resurgence of aqueous batteries will not depend solely on scientific progress; supportive policies and regulatory frameworks will be critical to their broader adoption. Governments and agencies increasingly emphasize energy storage safety, sustainability, and supply chain resilience. This opens doors for aqueous batteries, which use abundant, non‐critical materials (iron, zinc, manganese) and avoid reliance on cobalt and lithium.

Several policy levers could accelerate their adoption:

- Safety regulations: Stricter fire safety standards for grid and residential storage may favor aqueous systems over lithium‐ion.
- Sustainability incentives: Subsidies, tax credits, or preferential procurement policies for technologies using abundant and recyclable materials.
- Battery recycling policies: Frameworks that encourage closed‐loop recycling may highlight the simplicity of aqueous battery recycling compared to lithium‐ion.
- Strategic resource independence: Nations seeking to reduce dependence on lithium or cobalt imports may invest in aqueous battery supply chains.

## 6 Lesson‐Learned and Discussion for Future Battery Innovations

A historical perspective on battery development is not only valuable for contextualizing current progress but also essential for guiding future innovations. Importantly, no battery chemistry is intrinsically perfect: apparent breakthroughs often come with nontrivial trade‐offs, and improvements in one metric may be achieved at the expense of others. Therefore, beyond summarizing advances, it is equally necessary to explicitly acknowledge limitations and to distinguish fundamental progress from performance gains that may arise from favorable testing conditions or selective reporting. Many of the challenges we face today, such as metal dendrite growth, interfacial instability, and the pursuit of sustainable chemistries were encountered in different forms in earlier generations of batteries. By revisiting these trajectories, we can extract enduring principles, reinterpret once‐dismissed phenomena, and recognize the conditions under which abandoned technologies may return to relevance. In this sense, history functions both as a record of past successes and failures and as a reservoir of insights that can inspire new strategies. With this in mind, we highlight below several lessons from battery history and discuss how they may inform the future development of aqueous and non‐aqueous systems.

Cathode:

- Conversion Reactions Dominated Pre‐LIB Electrode Materials. Before the advent of intercalation chemistry, most rechargeable batteries relied on conversion‐type reactions. These materials often offered relatively high capacities, but their large structural changes during cycling resulted in poor reversibility and limited lifespan. With the growing demand for higher energy density, researchers have recently revisited conversion‐type electrodes (e.g., Li metal anode, Si, transition metal fluorides) as potential alternatives or complements to intercalation materials. This renewed interest suggests that past chemistries still hold untapped potential, particularly when combined with modern strategies such as nanostructuring and surface/interface engineering, which can mitigate some of the historical challenges. Nevertheless, it remains crucial to keep in mind the intrinsic drawbacks of conversion‐type electrodes compared with intercalation systems, especially regarding reversibility, efficiency, and long‐term durability.
- Stability Design of MnO_2_ Cathodes. Mn‐based compounds are highly attractive cathode materials owing to the multiple valence states of Mn, which can provide high capacities, and the abundance and low cost of Mn. However, several critical issues need to be addressed. In MnO_2_, Mn^4+^ (3d^3^, t_2g_^3^ e_g_^0^) is not Jahn–Teller active, leading to structural stability. Nevertheless, even a one‐electron reaction, which corresponds to a theoretical capacity of 308 mAh g^−1^, converts Mn^4+^ to Mn^3+^. The Mn^3+^ ion (3d^4^, t_2g_^3^e_g_^1^) has a singly occupied e_g_ orbital in a degenerate state, resulting in a strong Jahn–Teller effect and pronounced lattice distortion, thereby destabilizing the structure. In addition, Mn^3+^ is chemically unstable in acidic conditions and readily undergoes disproportionation, further compromising the overall stability. It is worth noting that in the spinel LiMn_2_O_4_, a widely studied Li‐ion cathode, the average Mn valence state lies between +3.5 and +4. Even so, this material still suffers from structural stability issues and has not become the mainstream choice for practical applications. Therefore, the intrinsic stability of Mn‐based cathodes must be taken seriously, particularly in highly polar aqueous electrolytes. How to improve their stability through fundamental materials design is of great importance. This also raises a critical question: are Mn‐based cathode systems truly suitable for long‐lifetime energy storage applications that require 15–20 years or even longer durability?

Anode:

- Zn‐Hg Amalgam and Its Influence on Metal Anodes. In the 19th century, Zn amalgamation with mercury was introduced to suppress corrosion and stabilize the zinc electrode. This alloying strategy not only improved the performance of Zn‐based batteries but also laid the foundation for thinking about how to manage reactive metal surfaces. In contemporary research, similar principles guide the stabilization of lithium and sodium metal anodes, where alloying with In, Sn, or Al helps mitigate dendrite growth and enhance cycling stability.
- Why Can Cd and Pb metal electrodes suppress dendrites. Notably, throughout their historical development, Pb and Cd metal electrodes demonstrated superior reversibility compared with other metallic anodes (e.g., Li, Zn, Na). Although the underlying mechanisms were not fully understood at the time, a key factor lies in the nature of the reaction products formed during cycling. In lead‐based systems, PbSO_4_, and in cadmium systems, Cd(OH)_2_, are either insoluble or only sparingly soluble in the electrolyte, unlike the readily soluble ionic products generated in zinc, lithium, or sodium systems. These insoluble products tend to form and remain in situ on the electrode surface, mitigating morphological changes and suppresses dendritic protrusions. Recently, Chao et al. demonstrated similar concept (solid‐to‐solid conversion) on Zn anode, enabling 91% Zn utilization in a Zn‐Ni battery with 80% capacity retention over 2000 cycles [171]. Revisiting such historical mechanisms with modern analytical techniques such as in situ microscopy and advanced interfacial characterization could yield valuable insights into strategies for controlling dendrite growth in contemporary lithium and zinc metal batteries. This principle of in situ forming insoluble, passivating conversion layers could be intentionally engineered in modern Zn or Li metal batteries using tailored electrolyte additives
- Zn Foil or Powder? In the earliest battery designs, such as the traditional dry cell, zinc metal foil served dual functions, acting both as the anode and as the battery container. This design, however, resulted in low Zn utilization and frequent leakage issues due to severe corrosion of the zinc casing. The advent of alkaline batteries brought a structural shift: zinc foil was replaced by zinc powder, which was packed inside the battery (Figure 4d). The use of alkaline electrolytes significantly mitigated side reactions of Zn metal, while the powder form increased surface area and improved zinc utilization. In contrast, over the past decade, research on Zn anodes has largely returned to zinc foil, often paired with mildly acidic electrolytes rather than alkaline ones. This shift raises a fundamental question: What is the optimal physical form of the zinc anode under different electrolyte conditions? Given that side reactions such as hydrogen evolution and corrosion are more pronounced in mildly acidic media compared to alkaline systems, using zinc powder in such environments appears ill‐advised. As a result, nearly all contemporary studies have opted for zinc foil. This, in turn, prompts further questions: How can zinc utilization be maximized when using foil? And when zinc foil is employed, what is the appropriate choice of current collector to ensure both electrochemical performance and structural compatibility? These questions deserve careful reflection for future research.

Electrolyte and SEI:

- Revisiting Zn‐based Electrolytes: From Acidic to Alkaline and Back. The development of Zn‐based batteries has seen a shift from early mild acid electrolytes (NH_4_Cl, ZnCl_2_) to alkaline systems (KOH), which significantly improved cycling stability and mitigated Zn corrosion. Interestingly, over the past decade, weakly acidic electrolytes, particularly ZnSO_4_ have regained attention, showing remarkably promising performance. Nonetheless, caution remains warranted. Zn corrosion and hydrogen evolution are inherently more pronounced in acidic media than in alkaline environments. Moreover, selecting suitable cathode materials under these conditions is still challenging, especially when comparing to the performance of Ni(OH)_2_ cathodes in alkaline electrolytes. Careful scrutiny of proposed mechanisms, interfacial chemistry, and long‐term stability is essential to distinguish genuine advances from potential artifacts of experimental design.
- SEI in Aqueous Batteries: The concept of the SEI originally emerged from lithium metal batteries and was long considered irrelevant in aqueous systems. For a long time, it was widely believed that aqueous battery systems do not form SEI. This is because water decomposition typically produces only gaseous products (H_2_ and O_2_), and any solid byproducts tend to dissolve readily, preventing the formation of a stable, passivating SEI as seen in organic electrolytes. However, with the development of high‐concentration electrolytes (or WiSE), SEI layers have been observed in aqueous batteries, composed mainly of inorganic compounds such as LiF or ZnF_2_. These interphases can effectively suppress hydrogen evolution and stabilize metal anodes. More recently, SEI formation has also been achieved in low‐concentration systems using specific additives or organic co‐solvents, extending the interphase concept beyond WiSE. This evolving understanding of SEI formation in aqueous systems has opened new pathways for electrolyte design. It bridges the gap between the passivation strategies used in non‐aqueous batteries and the challenges inherent to aqueous environments, pushing the boundaries of aqueous battery performance. Future research on aqueous batteries will likely focus on tailoring the interfacial chemistry through synergistic electrolyte/additive systems and understanding the dynamic evolution of SEI under practical cycling conditions.
- The Evolution of SEI Design: LiF as a “Harmful” Byproduct to a “Desirable” Component. In the early days of lithium battery research, the formation of lithium fluoride (LiF) in the SEI was considered detrimental, as it increased resistance and lowered ionic conductivity [172]. Over the past decade, however, LiF has been reinterpreted as a stabilizing component that enhances chemical stability, reduces solubility, and provides mechanical robustness. Today, electrolyte additives such as LiFSI or fluorinated solvents are deliberately employed to generate LiF‐rich SEIs. This evolution illustrates that scientific understanding progresses in a spiral rather than a straight line: we interpret experimental results based on the current state of knowledge, which can later prove to be incomplete or even incorrect. Similarly, we should approach claims with careful scrutiny: Does LiF truly represent a beneficial SEI component, or is its apparent advantage merely a consequence of the lithium salt and fluorinated solvents introduced into the electrolyte? In this context, critical thinking remains one of the most valuable qualities in scientific research.

Others:

- Development of Other Non‐active Battery Components: Current Collectors, Casings, and Separators. The design of non‐active components is equally critical in determining the practicality and cost of aqueous batteries. In alkaline environments, current collector technologies are relatively mature. But to resist corrosion, collectors and cell hardware are typically made of nickel or Ni‐plated substrates, which substantially raises costs and undermines the “low‐cost” advantage of aqueous systems. In strongly acidic environments, the challenge is even greater. Historically, very few battery chemistries have been successfully commercialized under such conditions. Lead–acid batteries and vanadium redox flow batteries are the most notable examples. Lead–acid batteries benefit from the intrinsic acid resistance of Pb‐based electrodes, while their casings are fabricated from inexpensive yet corrosion‐resistant polymers such as polypropylene, polyethylene, or acrylonitrile butadiene styrene. In contrast, vanadium redox flow batteries require much more stringent anti‐corrosion measures, including fluoropolymer such as polytetrafluoroethylene, polyvinylidene fluoride, perfluoroalkoxy alkanes, or carbon‐based coatings, which significantly increase system cost. Even for mild acidic electrolytes such as ZnSO_4_ solutions, the choice of collectors remains challenging. Carbon, stainless steel, and titanium are often used in laboratories, but all come with considerable expense.
- Separators represent another critical bottleneck. Most laboratory‐scale aqueous batteries rely on glass fiber separators, which offer excellent wettability and ionic conductivity. However, their poor mechanical toughness makes them incompatible with large‐scale roll‐to‐roll fabrication processes, such as those used in LIBs. In addition, their significant thickness increases electrolyte consumption and reduces overall energy density. Future research should therefore explore thinner, stronger, and more scalable alternatives, such as modified polyolefin membranes, cellulosic papers, or ceramic‐coated composites. Taken together, these considerations highlight the need for a holistic evaluation of cost and manufacturability. Claims of “low cost” for aqueous batteries should be made cautiously, taking into account not only electrode and electrolyte materials but also collectors, casings, and separators.
- Besides, bipolar stacking, which enables internal series connections through bipolar plates, represents a promising route to construct high‐voltage aqueous battery modules while reducing external wiring and interconnect complexity. Compared with conventional liquid‐electrolyte Li‐ion packs that rely on extensive external connections and multi‐cell management to reach high voltages, the nonflammable nature of aqueous electrolytes provides greater tolerance for compact module integration. Nevertheless, practical bipolar implementations remain chemistry‐ and architecture‐dependent, as they demand robust inter‐cell sealing to suppress shunt currents and must address gas generation and swelling, particularly in Zn‐based systems.
- Beyond rediscovery to boundary pushing in scientific research. As early as reported in 1998, studies on MnO_2_ cathodes in mildly acidic ZnSO_4_ electrolytes had already established a clear correlation between capacity fading, Mn dissolution, and the formation of basic zinc sulfate by products, and further showed that adding a suitable concentration of MnSO_4_ could markedly improve cycling stability [173]. Nearly twenty years later, when ZnSO_4_ based electrolytes re‐entered the mainstream of Zn‐MnO_2_ research, many works essentially reproduced the same phenomenology with more advanced characterization and computation, but without a fundamentally new picture of the degradation chemistry. Revisiting classical systems with new tools is valuable, yet genuine innovation should consciously stand on prior insights, move beyond reconfirming known mechanisms, and push toward sharper mechanistic questions and more application relevant metrics, rather than simply reheating well explored chemistries.
- The Rise and Fall of Battery Chemistries: What Determines a Comeback? Battery development has never followed a linear path of progress. Sodium‐ion batteries, for example, were widely studied in the 1970s and 1980s before being overshadowed by lithium‐ion. Decades later, concerns about sustainability and resource availability have brought them back to the forefront. This cyclical pattern raises the question: under what conditions could aqueous batteries stage a similar comeback? Historical precedents suggest that shifts in raw material supply, safety regulations, or new application niches may create the right environment for revival.

The history of battery chemistry is not linear but cyclical. The revival of aqueous systems today is driven by a new set of market and societal forces (i.e., safety, sustainability, and cost for stationary storage), that mirror the very forces that led to their initial success, demonstrating that technological progress is often a process of recontextualization and refinement.

## 7 Conclusion

The journey of aqueous batteries, from their pioneering role in the 19th century to their current resurgence, underscores their enduring relevance in the energy storage landscape. While their historical limitations in energy density and cycling stability ceded ground to lithium‐ion batteries, recent innovations, such as water‐in‐salt and localized high‐concentration electrolytes, advanced electrode designs, and application‐specific developments, have significantly narrowed these gaps. Aqueous batteries are not poised to displace lithium‐ion systems in high‐energy‐density applications like electric vehicles or portable electronics, but are carving out in grid‐scale storage, uninterruptible power supplies for data centers, and decentralized energy systems where safety, cost, and sustainability are paramount. Historical lessons continue to inform modern strategies, reminding us that past challenges often hold the seeds of future solutions. With supportive policies emphasizing safety, recyclability, and supply chain resilience, aqueous batteries are well‐positioned to complement lithium‐ion technologies, contributing to a diversified and sustainable energy storage ecosystem. Continued research into scalable electrolyte designs, durable electrode materials, and cost‐effective manufacturing will be essential to realize their full potential, ensuring aqueous batteries play a pivotal role in powering a greener, more resilient future.

## Conflicts of Interest

The authors declare no conflicts of interest.

## Data Availability

The data that support the findings of this study are available on request from the corresponding author. The data are not publicly available due to privacy or ethical restrictions.
